# Supplementary material for: Proteomic Analysis of Mucosal and Systemic Responses to SARS-CoV-2 Antigen
Source: Vaccines (Basel). 2023 Feb 2;11(2):334. doi: 10.3390/vaccines11020334 (PMC9960779; doi:10.3390/vaccines11020334)
Supplement: Supplementary file 1 [file vaccines-11-00334-s001.zip › Figure S1.pdf]

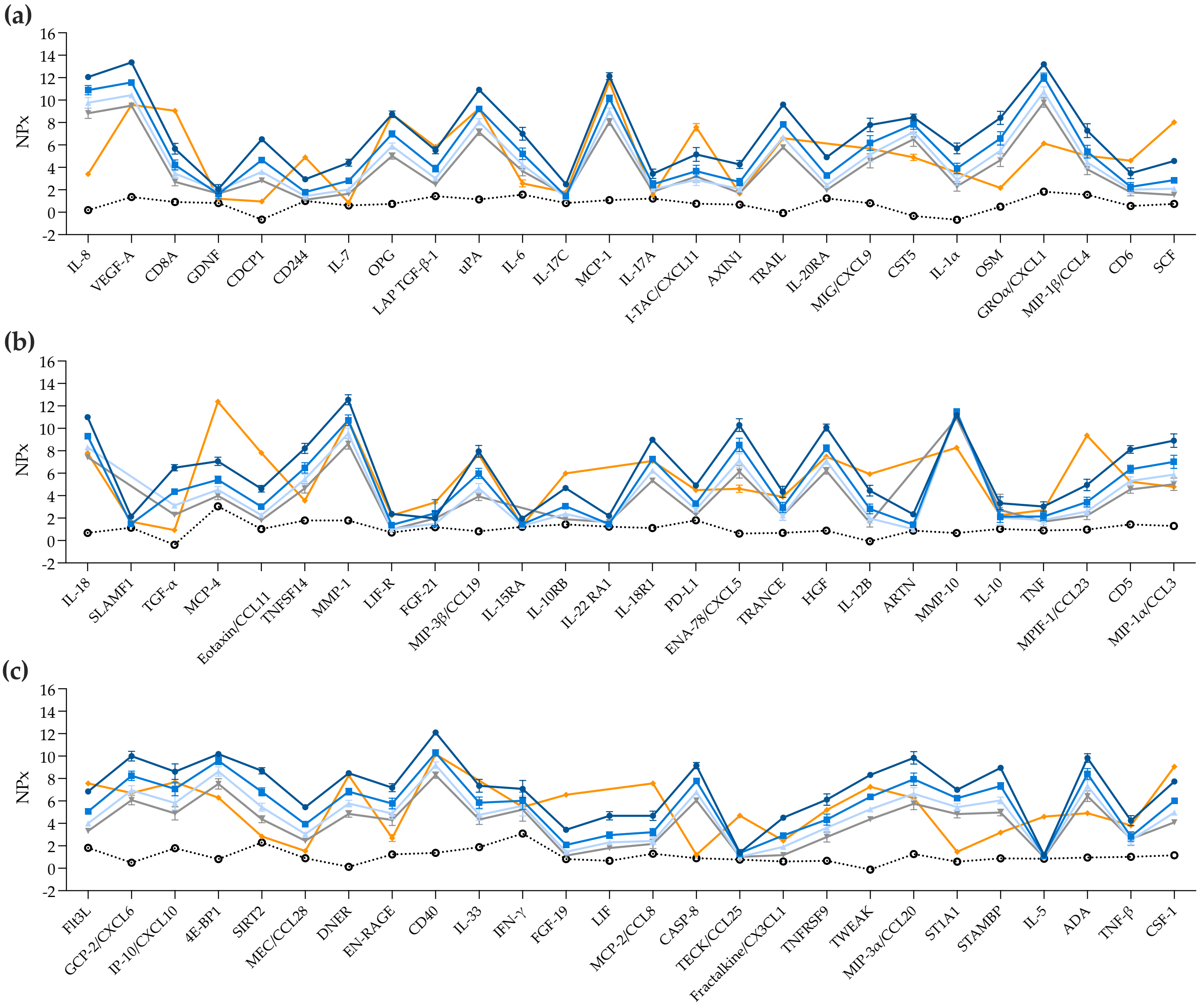

Figure S1. Optimization of nasopharyngeal (NP) secretions dilution for Olink assay. Secretions eluted from NP swabs were analyzed on the Olink platform undiluted (●) and at three dilutions, 1:4 (■), 1:8 (▲) and 1:16(▼). Normalized protein expression (NPx) data for NP secretions and undiluted plasma (◆) above the lower limit of detection (○) are shown as mean (± SEM) of all participants. One technical replicate was analyzed per sample, per dilution for each participant.
