# Supplementary material for: Proteomic Analysis of Mucosal and Systemic Responses to SARS-CoV-2 Antigen
Source: Vaccines (Basel). 2023 Feb 2;11(2):334. doi: 10.3390/vaccines11020334 (PMC9960779; doi:10.3390/vaccines11020334)
Supplement: Supplementary file 1 [file vaccines-11-00334-s001.zip › Figure S2.pdf]

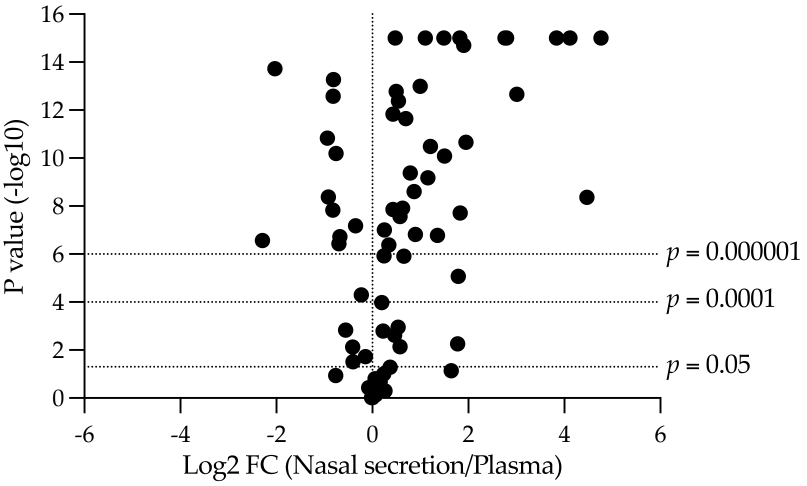

Figure S2. Comparison of systemic and nasopharyngeal (NP) secretion proteomic profiles. Plasma and NP swabs were obtained from each study participant. Plasma was analyzed undiluted. NP secretions were collected from swabs and analyzed undiluted. Volcano plots comparing normalized protein expression (NPx) levels in NP secretions to plasma. FC: fold change. Analytes above horizontal dotted line corresponding to a *p* value of 0.05 are significantly modulated. Vertical dotted line is set at a Log2FC = 0.
