# Supplementary material for: Proteomic Analysis of Mucosal and Systemic Responses to SARS-CoV-2 Antigen
Source: Vaccines (Basel). 2023 Feb 2;11(2):334. doi: 10.3390/vaccines11020334 (PMC9960779; doi:10.3390/vaccines11020334)
Supplement: Supplementary file 1 [file vaccines-11-00334-s001.zip › Figure S4.pdf]

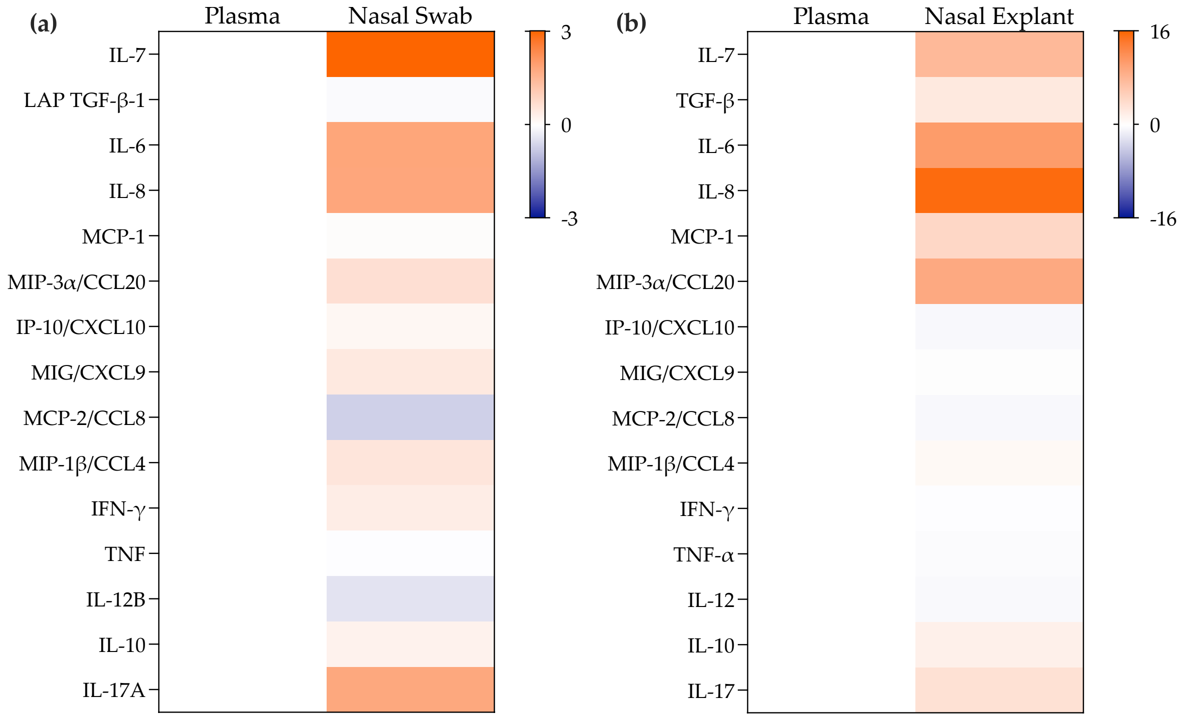

Figure S4. Relative cytokine expression in the nasal compartment to plasma. Plasma and nasal tissue and swab were obtained from each participant. Heatmaps represent upregulated (orange), or downregulated (blue) common cytokines measured via Luminex and Olink platforms. Differences between cytokine levels in (**a**) nasal secretions and plasma measured by Olink, and in (**b**) nasal tissue and plasma measured by Luminex, are shown as Log2. For each participant, biological triplicates were analyzed by Luminex, and single replicates by Olink technology.
