# Supplementary material for: Proteomic Analysis of Mucosal and Systemic Responses to SARS-CoV-2 Antigen
Source: Vaccines (Basel). 2023 Feb 2;11(2):334. doi: 10.3390/vaccines11020334 (PMC9960779; doi:10.3390/vaccines11020334)
Supplement: Supplementary file 1 [file vaccines-11-00334-s001.zip › Figure S5.pdf]

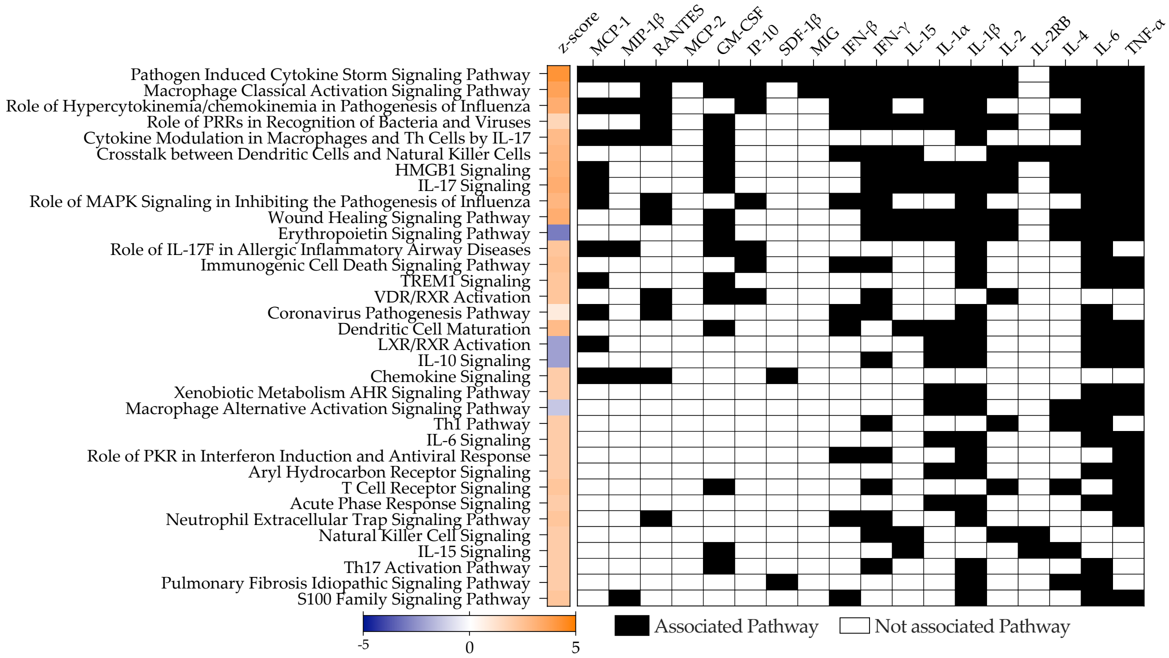

Figure S5. Canonical pathways significantly enriched in oral explant culture supernatants in comparison to nasal tissue. The activation z-scores determined by directionality (overabundance in orange and underabundance in blue) and number of cytokine/chemokine are represented by a heat map. The proteins associated to each pathway are shown in black cells. The pathways included had a minimum of two analytes associated and a $p < 0.05$ (right-tailed Fisher exact test).
