# Supplementary material for: Proteomic Analysis of Mucosal and Systemic Responses to SARS-CoV-2 Antigen
Source: Vaccines (Basel). 2023 Feb 2;11(2):334. doi: 10.3390/vaccines11020334 (PMC9960779; doi:10.3390/vaccines11020334)
Supplement: Supplementary file 1 [file vaccines-11-00334-s001.zip › Figure S6.pdf]

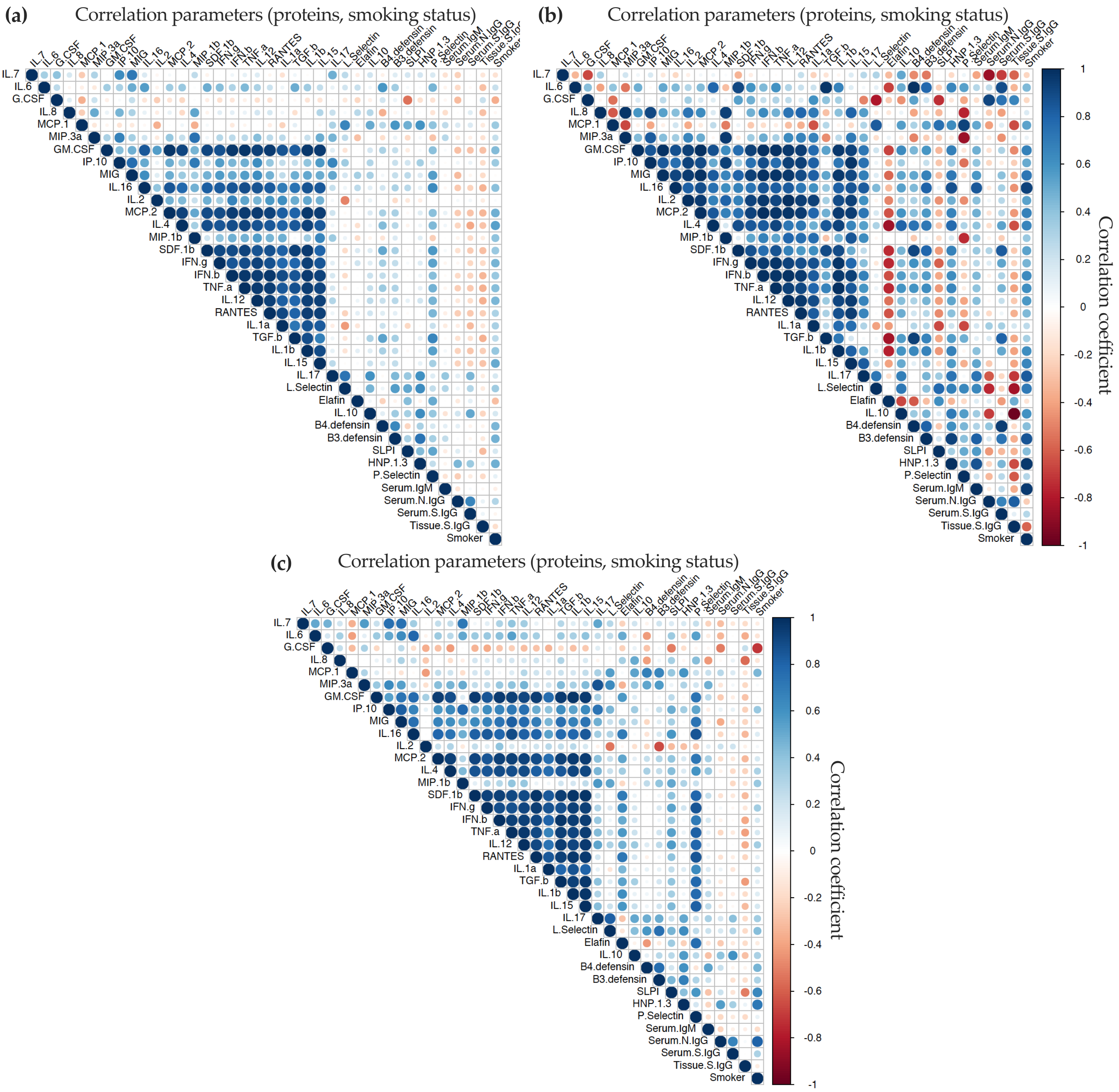

Figure S6. Correlogram of responses elicited systemically. Correlations between concentrations of plasma cytokines/chemokines, serum and tissue Ab levels and smoking-status for all participants (**a**), vaccine-naïve (**b**) or BioNTech/Pfizer-vaccinated participants (**c**) were analyzed by a correlation matrix. Positive Pearson correlations coefficients are displayed in blue and negative correlations in red color. The color intensity indicated in the side bar is proportional to the correlation coefficient. The size of the circle is proportional to the statistical significance of the correlation.
