# Supplementary material for: Proteomic Analysis of Mucosal and Systemic Responses to SARS-CoV-2 Antigen
Source: Vaccines (Basel). 2023 Feb 2;11(2):334. doi: 10.3390/vaccines11020334 (PMC9960779; doi:10.3390/vaccines11020334)
Supplement: Supplementary file 1 [file vaccines-11-00334-s001.zip › Table S1.pdf]

Table S1. Baseline characteristics of COVAB study participants

Characteristic		COVAB study (n=18)
Gender		
	Female	7 (39%)
	Male	11 (61%)
Age (years), mean (SD)		40.2 (14.8)
BMI, mean (SD)		27.3 (5.6)
Living with HIV		0
Currently smoking		
	No	13 (72%)
	Yes	5 (28%)
Diabetes		
	No	18 (100%)
	Yes	0 (0%)
Hypertension		
	No	15 (83%)
	Yes	3 (17%)
Asthma and/or COPD[1]		
	No	12 (67%)
	Yes	6 (33%)
Previous positive COVID-19 test[2]		
	No	14 (78%)
	Yes	4 (22%)
COVID-19 vaccinated		
	No	6 (33%)
	Yes	12 (67%)
COVID-19 vaccine[3]		
	BioNTech/Pfizer	11 (92%)
	J&J	1 (8%)
Time since vaccination (weeks), mean (SD)		19.8 (14.9)
Vaccinated and infected[3,4]		
	No	8 (67%)
	Yes	4 (33%)

[1] COPD: chronic obstructive pulmonary disease.
[2] Positive test at least 4 months before surgery.
[3] % calculated among number of vaccinated participants.
[4] Vaccinated individuals having tested positive for COVID-19 at least 4 months before surgery.
