# Supplementary material for: Proteomic Analysis of Mucosal and Systemic Responses to SARS-CoV-2 Antigen
Source: Vaccines (Basel). 2023 Feb 2;11(2):334. doi: 10.3390/vaccines11020334 (PMC9960779; doi:10.3390/vaccines11020334)
Supplement: Supplementary file 1 [file vaccines-11-00334-s001.zip › Table S2.pdf]

Table S2. List of differentially expressed proteins in nasopharyngeal (NP) swabs and plasma[1].

Protein	Definition[2]	NP Swab *vs.* Plasma FC	*P* value
IL-8	Homo sapiens interleukin 8	3.541	< 0.0001
VEGF-A	Homo sapiens vascular endothelial growth factor-A	1.394	< 0.0001
CD8A	Homo sapiens T-cell surface glycoprotein CD8 alpha chain	0.627	< 0.0001
GDNF	Homo sapiens glial cell line-derived growth factor	3.432	0.0054
CDCP1	Homo sapiens CUB domain containing protein 1	6.774	< 0.0001
CD244	Homo sapiens natural killer cell receptor 2B4	0.565	< 0.0001
IL-7	Homo sapiens interleukin 7	8.055	< 0.0001
OPG	Homo sapiens osteoprotegerin	1.001	0.9691
LAP TGF-β-1	Homo sapiens latency-associated peptide and mature TGF-beta 1	0.947	0.3694
uPA	Homo sapiens uterine-specific proline-rich acidic protein	1.181	< 0.0001
IL-6	Homo sapiens interleukin 6	3.554	< 0.0001
IL-17C	Homo sapiens interleukin 17C	1.495	0.0072
MCP-1	Homo sapiens monocyte chemotactic protein-induced protein 1	1.042	0.1547
IL-17A	Homo sapiens interleukin 17A	3.460	< 0.0001
I-TAC/CXCL11	Homo sapiens interferon inducible T-cell alpha chemokine	0.679	0.0015
AXIN1	Homo sapiens axis inhibition protein 1	2.564	< 0.0001
TRAIL	Homo sapiens TNF-related apoptosis-inducing ligand	1.455	< 0.0001
IL-20RA	Homo sapiens Interleukin-20 receptor subunit alpha	N/A	N/A
MIG/CXCL9	Homo sapiens monokine-induced by interferon gamma	1.376	0.0024
CST5	Homo sapiens cystatin-5	1.727	< 0.0001
IL-1α	Homo sapiens interleukin 1 alpha	N/A	N/A
OSM	Homo sapiens oncostatin-M	3.857	< 0.0001
GROα/CXCL1	Homo sapiens growth regulated protein alpha	2.146	< 0.0001
MIP-1β/CCL4	Homo sapiens macrophage inflammatory protein 1-beta	1.447	0.0011
CD6	Homo sapiens T-cell differentiation antigen CD6	0.757	0.0307
SCF	Homo sapiens stem cell factor	0.569	< 0.0001
IL-18	Homo sapiens interleukin 18	1.411	< 0.0001
SLAMF1	Homo sapiens signalling lymphocytic activation molecule family member 1	0.589	0.1150
TGF-α	Homo sapiens protransforming growth factor alpha	6.952	< 0.0001
MCP-4/CCL13	Homo sapiens monocyte chemoattractant protein 4	0.571	< 0.0001
Eotaxin/CCL11	Homo sapiens eotaxin	0.591	< 0.0001
TNFSF14	Homo sapiens tumor necrosis factor receptor superfamily member 14	2.311	< 0.0001
MMP-1	Homo sapiens matrix metalloproteinase-1	1.168	0.0016
LIF-R	Homo sapiens leukemia inhibitory factor receptor	1.057	0.5694
FGF-21	Homo sapiens fibroblast growth factor 21	0.204	< 0.0001
MIP-3β/CCL19	Homo sapiens macrophage inflammatory protein 3 beta	1.037	0.6194
IL-15RA	Homo sapiens interleukin-15 receptor subunit alpha	22.173	< 0.0001
IL-10RB	Homo sapiens interleukin-10 receptor subunit beta	0.783	< 0.0001
IL-22RA1	Homo sapiens interleukin-22 receptor subunit alpha-1	N/A	N/A
IL-18R1	Homo sapiens interleukin-18 receptor 1	1.268	< 0.0001
PD-L1	Homo sapiens programmed death ligand 1	1.093	0.1619
ENA-78/CXCL5	Homo sapiens epithelial neutrophil activating peptide 78	2.231	< 0.0001
TRANCE	Homo sapiens TNF-related activation-induced cytokine	1.048	0.7341
HGF	Homo sapiens hepatocyte growth factor	1.348	< 0.0001
IL-12B	Homo sapiens interleukin-12 subunit beta	0.751	0.0075
ARTN	Homo sapiens artemin	N/A	N/A
MMP-10	Homo sapiens matrix metalloproteinase-10	1.349	< 0.0001
IL-10	Homo sapiens interleukin 10	1.201	0.4992
TNF	Homo sapiens tumor necrosis factor	0.988	0.9442
MPIF-1/CCL23	Homo sapiens myeloid progenitor inhibitory factor 1	0.529	< 0.0001
CD5	Homo sapiens T-cell surface glycoprotein CD5	1.550	< 0.0001
MIP-1α/CCL3	Homo sapiens macrophage inflammatory protein 1-alpha	1.861	< 0.0001
Flt3L	Homo sapiens Fms-related tyrosine kinase 3 ligand	0.903	0.0189
GCP-2/CXCL6	Homo sapiens gamma-tubulin complex component 2	1.489	< 0.0001
IP-10/CXCL10	Homo sapiens 10 kDa interferon gamma-induced protein	1.122	0.2051
4E-BP1	Homo sapiens eukaryotic translation initiation factor 4E-binding protein 1	1.618	< 0.0001
SIRT2	Homo sapiens NAD-dependent protein deacetylase sirtuin-2	17.336	< 0.0001
MEC/CCL28	Homo sapiens mucosae-associated epithelial chemokine	3.742	< 0.0001
DNER	Homo sapiens delta and Notch-like epidermal growth factor-related receptor	1.024	0.4833

EN-RAGE	Homo sapiens extracellular newly identified RAGE (Receptor for Advanced Glycosylation End products)-binding protein	2.839	< 0.0001
CD40	Homo sapiens B-cell surface antigen CD40	1.190	< 0.0001
IL-33	Homo sapiens interleukin 33	N/A	N/A
IFN-γ	Homo sapiens interferon-gamma	1.294	0.0524
FGF-19	Homo sapiens fibroblast growth factor 19	0.522	< 0.0001
LIF	Homo sapiens leukemia inhibitory factor	N/A	N/A
MCP-2/CCL8	Homo sapiens monocyte chemoattractant protein 2	0.617	< 0.0001
CASP-8	Homo sapiens caspase-8	14.314	< 0.0001
TECK/CCL25	Homo sapiens thymus-expressed chemokine	0.245	< 0.0001
Fractalkine/CX3CL1	Homo sapiens fractalkine	1.828	< 0.0001
TNFRSF9	Homo sapiens tumor necrosis factor receptor superfamily member 9	1.179	0.0971
TWEAK	Homo sapiens TNF-related weak inducer of apoptosis	1.145	0.0001
MIP-3α/CCL20	Homo sapiens macrophage inflammatory protein 3 alpha	1.573	< 0.0001
ST1A1	Homo sapiens aryl sulfotransferase 1	27.131	< 0.0001
STAMBP	Homo sapiens STAM (Signal Transducing Adapter Molecule)-binding protein	2.812	< 0.0001
IL-5	Homo sapiens interleukin 5	3.118	0.0720
ADA	Homo sapiens adenosine deaminase	2.000	< 0.0001
TNF-β	Homo sapiens tumor necrosis factor beta	1.044	0.7420
CSF-1	Homo sapiens macrophage colony-stimulating factor 1	0.855	< 0.0001

[1]Significance determined by unpaired *t* test with a Benjemini-Hochberg multiple testing correction ($P < 0.05$).

[2]UnitProt definitions.

FC: Fold change.

N/A: not applicable, protein not detectable in plasma.
