# Supplementary material for: Proteomic Analysis of Mucosal and Systemic Responses to SARS-CoV-2 Antigen
Source: Vaccines (Basel). 2023 Feb 2;11(2):334. doi: 10.3390/vaccines11020334 (PMC9960779; doi:10.3390/vaccines11020334)
Supplement: Supplementary file 1 [file vaccines-11-00334-s001.zip › Table S3.pdf]

Table S3. List of differentially expressed proteins in nasal explant culture supernatants and plasma[1].

Protein	Definition[2]	Tissue *vs.* Plasma FC	*P* value
IL-7	Homo sapiens interleukin 7	200.867	< 0.0001
IL-6	Homo sapiens interleukin 6	1,674.125	0.0066
G-CSF	Homo sapiens granulocyte colony-stimulating factor receptor	160.322	< 0.0001
IL-8	Homo sapiens interleukin 8	47,626.175	0.0117
MCP-1	Homo sapiens monocyte chemotactic protein-induced protein 1	22.078	0.0004
MIP-3α/CCL20	Homo sapiens macrophage inflammatory protein 3 alpha	632.631	< 0.0001
GM-CSF	Homo sapiens granulocyte-macrophage colony-stimulating factor	0.705	0.0035
IP-10/CXCL10	Homo sapiens 10 kDa interferon gamma-induced protein	0.650	0.2552
MIG/CXCL9	Homo sapiens monokine-induced by interferon gamma	0.875	0.5293
IL-16	Homo sapiens interleukin 16	1.338	0.0160
IL-2	Homo sapiens interleukin 2	3.427	< 0.0001
MCP-2/CCL8	Homo sapiens monocyte chemoattractant protein 2	0.654	< 0.0001
IL-4	Homo sapiens interleukin 4	0.815	0.0004
MIP-1β/CCL4	Homo sapiens macrophage inflammatory protein 1 beta	1.596	0.1726
SDF-1β	Homo sapiens stromal cell-derived factor 1 beta	0.704	0.0112
IFN-γ	Homo sapiens interferon-gamma	0.953	0.5822
IFN-β	Homo sapiens interferon-beta	0.594	< 0.0001
TNF-α	Homo sapiens tumor necrosis factor alpha	0.775	0.0870
IL-12	Homo sapiens interleukin 12	0.691	0.0066
RANTES/CCL5	Homo sapiens chemokine (C-C motif) ligand 5	0.104	0.0005
IL-1α	Homo sapiens interleukin 1 alpha	6.827	0.1677
TGF-β	Homo sapiens transforming growth factor beta	5.607	0.2074
IL-1β	Homo sapiens interleukin 1 beta	1.045	0.8456
IL-15	Homo sapiens interleukin 15	0.691	0.0050
IL-17	Homo sapiens interleukin 17	10.115	< 0.0001
L-Selectin	Homo sapiens leukocyte-endothelial cell adhesion molecule 1	0.001	< 0.0001
Elafin	Homo sapiens elastase-specific inhibitor,	5.985	0.0043
IL-10	Homo sapiens interleukin 10	3.197	< 0.0001
HBD4	Homo sapiens beta defensin 4	5.441	< 0.0001
HBD3	Homo sapiens beta defensin 3	4.327	< 0.0001
SLP1	Homo sapiens secretory leukocyte protease inhibitor 1	25.528	0.0726
HNP-1-3	Homo sapiens alpha-defensin/human neutrophil peptide 1-3	5.448	< 0.0001
P-Selectin	Homo sapiens leukocyte-endothelial cell adhesion molecule 3	6.840	< 0.0001

[1]Significance determined by unpaired *t* test with a Benjemini-Hochberg multiple testing correction ($P < 0.05$).
[2]UnitProt definitions.
FC: Fold change.
