# Supplementary material for: Proteomic Analysis of Mucosal and Systemic Responses to SARS-CoV-2 Antigen
Source: Vaccines (Basel). 2023 Feb 2;11(2):334. doi: 10.3390/vaccines11020334 (PMC9960779; doi:10.3390/vaccines11020334)
Supplement: Supplementary file 1 [file vaccines-11-00334-s001.zip › Table S4.pdf]

Table S4. List of differentially expressed proteins in nasal and oral explant culture supernatants[1].

Protein	Definition[2]	Oral *vs.* Nasal FC	*P* value
IL-7	Homo sapiens interleukin 7	0.734	0.2461
IL-6	Homo sapiens interleukin 6	1.061	0.5790
G-CSF	Homo sapiens granulocyte colony-stimulating factor receptor	0.925	0.8097
IL-8	Homo sapiens interleukin 8	0.328	0.1297
MCP-1	Homo sapiens monocyte chemotactic protein-induced protein 1	1.350	0.4771
MIP-3α/CCL20	Homo sapiens macrophage inflammatory protein 3 alpha	0.792	0.1788
GM-CSF	Homo sapiens granulocyte-macrophage colony-stimulating factor	2.374	0.0050
IP-10/CXCL10	Homo sapiens 10 kDa interferon gamma-induced protein	2.431	0.2313
MIG/CXCL9	Homo sapiens monokine-induced by interferon gamma	1.209	0.3112
IL-16	Homo sapiens interleukin 16	1.054	0.1436
IL-2	Homo sapiens interleukin 2	2.025	0.0260
MCP-2/CCL8	Homo sapiens monocyte chemoattractant protein 2	1.067	0.0367
IL-4	Homo sapiens interleukin 4	1.018	0.1120
MIP-1β/CCL4	Homo sapiens macrophage inflammatory protein 1 beta	1.128	0.7016
SDF-1β	Homo sapiens stromal cell-derived factor 1 beta	1.081	0.6216
IFN-γ	Homo sapiens interferon-gamma	1.149	0.3350
IFN-β	Homo sapiens interferon-beta	1.196	0.0176
TNF-α	Homo sapiens tumor necrosis factor alpha	1.691	0.0241
IL-12	Homo sapiens interleukin 12	1.788	0.0359
RANTES/CCL5	Homo sapiens chemokine (C-C motif) ligand 5	13.346	0.0411
IL-1α	Homo sapiens interleukin 1 alpha	20.116	0.0618
TGF-β	Homo sapiens transforming growth factor beta	0.505	0.5063
IL-1β	Homo sapiens interleukin 1 beta	3.937	0.0175
IL-15	Homo sapiens interleukin 15	1.520	0.0090
IL-17	Homo sapiens interleukin 17	0.422	0.0128
L-Selectin	Homo sapiens leukocyte-endothelial cell adhesion molecule 1	0.379	0.0291
Elafin	Homo sapiens elastase-specific inhibitor,	1.519	0.2081
IL-10	Homo sapiens interleukin 10	0.651	0.0853
HBD4	Homo sapiens beta defensin 4	0.839	0.0110
HBD3	Homo sapiens beta defensin 3	0.633	0.1863
SLP1	Homo sapiens secretory leukocyte protease inhibitor 1	0.407	0.1414
HNP-1-3	Homo sapiens alpha-defensin/human neutrophil peptide 1-3	0.459	0.0512
P-Selectin	Homo sapiens leukocyte-endothelial cell adhesion molecule 3	0.534	0.0755

[1]Significance determined by unpaired *t* test with a Benjemini-Hochberg multiple testing correction ($P < 0.05$).
[2]UnitProt definitions.
FC: Fold change.
